# Effects of balance training on static and dynamic balance performance in healthy children: role of training duration and volume

**DOI:** 10.1186/s13104-021-05873-5

**Published:** 2021-12-23

**Authors:** Thomas Muehlbauer

**Affiliations:** Division of Movement and Training Sciences/Biomechanics of Sport, University of Duisburg-Essen, Gladbecker Str. 182, 45141 Essen, Germany

**Keywords:** Postural control, Childhood, Intervention, Dose–response relationship

## Abstract

**Objective:**

Improvements in balance performance through balance training programs in children have been reported in several studies. However, the influence of balance training modalities (e.g., training period, frequency, volume) on the training effectiveness have not yet been studied. To address this shortfall, the present study investigated the effects of balance training duration and volume (i.e., 240 min during 4 weeks versus 360 min during 6 weeks) on measures of static and dynamic balance performance in healthy children (*N* = 29) aged 10 years.

**Results:**

Irrespective of balance training duration and volume, significant pre- to post-test improvements were found for variables of static (i.e., one-legged stance on foam ground, reduced number of floor contacts: *p* = .041, *η*_p_^2^ = .15) and dynamic (i.e., Lower Quarter Y Balance test, increased anterior reach distance: *p* = .038, *η*_p_^2^ = .15) balance performance but no group × test interactions were detected. These findings indicate that balance training is effective to improve static and dynamic balance performance in healthy children, but the effectiveness seems unaffected by the applied training duration and volume.

*Trial Registration* Current Controlled Trials ISRCTN75170753 (retrospectively registered at 12th April, 2021).

## Introduction

Balance training (BT) is an effective method to improve various types of balance performance in youth [1–4]. For example, Dobrijevic et al. [4] detected significant improvements in stance time following 12 weeks of BT (2 sessions/week; 10 min/session) in 7–8-year-old children. Moreover, Granacher et al. [2] found significantly reduced postural sway in adolescents (~ 18 years) after 4 weeks of BT (3 sessions/week, 30 min each). Lastly, Schedler et al. [3] reported that 5 weeks of BT (2–3 times per week with a total of 135 min per week) resulted in significantly improved postural sway and gait velocity in children (7.5 ± 0.5 years) and adolescents (14.7 ± 0.5 years).

However, a closer look at these and other studies shows that the used design of the training load varies greatly. More specifically, BT duration ranged between 4 and 12 weeks, BT frequency between 2 and 7 times per week, and BT volume between 10 and 36 sessions [1]. Thus, it remains unclear which type of load design is particularly effective for BT to improve balance performance in youth. A first clue to answer this question is provided by the systematic review with meta-analysis on the effects and dose–response relationships of BT in youth by Gebel and colleagues [1]. They analysed 17 studies and quantified the effect sizes for individual BT modalities. With regard to BT duration, a distinction was made between 4, 5, 6, and 12 weeks and effect sizes of 0.78, 0.51, 0.32, and 1.40 were calculated, respectively. This finding shows that an increase in BT duration is not necessarily associated with a linear increase in BT effectiveness, but appears to follow a J-shaped distribution. However, this rating is only valid to a limited extent, as Gebel et al. [1] performed an indirect (between-study) comparison of BT regimes with short versus long BT durations but no direct (within-study) comparison (i.e., a study including two groups that conducted BT with different program durations). Regarding BT volume, Gebel et al. [1] excluded training modalities such as ‘number of exercises’, ‘duration of a set’, ‘number of repetitions’, and ‘duration of an exercise’ from their dose–response quantifications due to the limited data availability.

Therefore, the aim of this study was to directly compare the effectiveness of 4 weeks (i.e., 240 min of training) versus 6 weeks (i.e., 360 min of training) of BT on children's static and dynamic balance performance. First, it was assumed that both BT programs will result in improvements in balance. Second, it was expected that a longer and more voluminous exposure to the balance demanding training stimuli will lead to greater improvements.

## Main text

### Methods

#### Participants

An a priori power analysis using G*Power 3.1.9.2 [5] with the following input parameters was performed: *f* = 0.25, *α* = 0.05, 1-*β* = 0.80, groups (*n* = 2), measurements (*n* = 2), correlation between measurements (*r* = 0.6), drop-out rate per group (10%) and revealed a total sample size of *N* = 28 participants. Therefore, thirty-four healthy children were included and randomly assigned to a BT-group with a training duration/volume of 4 weeks (BT-4wk including 240 min of training) or 6 weeks (BT-6wk including 360 min of training). The inclusion criteria for participation were (1) willingness to participate; (2) ability to participate in group activities (i.e., free of visual/auditory impairments). Children were excluded from participation if they (1) had a musculoskeletal or neurological disorder during the last three months prior to the beginning of the study; (2) had other medical conditions that could have affected their ability to execute the assessments and BT exercises; (3) performed the pretest or posttest only. Two children of the BT-4wk and three children of the BT-6wk performed either only the pretest or the posttest and were not included in the data analysis. Participants’ characteristics by group are shown in Table 1. The examiners were blinded to group allocation and the participants were only aware of their own training program (i.e., BT-4wk or BT-6wk), but did not know how other participants trained.

**Table 1:** Characteristics of the study participants (*N* = 29) by group

Outcome	BT-4wk (*n* = 15)	BT-6wk (*n* = 14)
Age [years]	10.1 ± 0.4	10.4 ± 0.5
Sex [f/m]	6/9	8/6
Body height [cm]	152.8 ± 4.3	146.6 ± 6.2
Body mass [kg]	45.7 ± 11.6	38.0 ± 5.4
Body mass index [kg/m^2^]	19.5 ± 4.6	17.6 ± 1.6
Leg length [cm]	80.7 ± 3.8	80.6 ± 5.7
Leg dominance [l/r]	2/13	0/14

Data are presented as mean value ± standard deviation

*BT* balance training, *f* female, *l* left, *m* male, *r* right

#### Assessment of static balance

Static balance was assessed using the one-legged stance (OLS). Participants had to stand with their non-dominant leg (i.e., stance leg when kicking a ball) without shoes, while the other leg was flexed to approximately 90°. Further, they had to place their hands on the hips and to fixate a cross marked at eye level on a nearby wall. The participants were instructed to stand with eyes open (EO) for maximal 60 s without touching the ground; first on firm (FI) ground followed by standing on foam (FO) ground (i.e., balance pad). In both conditions, the number of floor contacts (*n*; i.e., when balance was lost and the contralateral leg touched the floor) during the test duration was noted and used as outcome measure. Thus, the lower the value, the better the static balance performance.

#### Assessment of dynamic balance

Dynamic balance was assessed using the Lower Quarter Y-Balance Test (YBT-LQ; Functional Movement Systems®, Chatham, USA) that consisted of a centralized stance platform to which three pipes were attached in anterior (AT), posteromedial (PM), and posterolateral (PL) directions. The participants were asked to stand on the centralized platform on their non-dominant leg without shoes while reaching as far as possible in AT, PM, and PL directions with the other leg by moving reach indicators attached to the pipes without losing balance. Each participant performed three practice trials followed by three data-collection trials per reach direction with arm movements permitted during test execution. As recommended by Plisky et al. [6], a trial was classified as invalid if the participants (1) lost their balance (i.e., stepped with the reach leg on the floor); (2) lifted the stance leg from the centralized platform; (3) stepped on top of the reach indicator for support; or (4) kicked the reach indicator. The maximal reach distance (cm) per reach direction was normalized to the participant’s leg length (LL, i.e., distance from the distal end of the anterior superior iliac spine to the most distal point of the medial malleolus) using the following equation: normalized reach distance (%LL) = maximal reach distance / LL × 100. Further, a composite score (CS) was calculated using the following formula: CS (%LL) = ((AT + PM + PL) ∕ (LL × 3)) × 100. Overall, the higher the value, the better the dynamic balance performance.

#### Balance training programs

Standardized BT programs were conducted for 4 and 6 weeks respectively with two sessions per week (30 min per session) at a school gym. This led to a total BT volume of 240 min (BT-4wk) and 360 min (BT-6wk), respectively. Graduate students supervised the BT programs and provided verbal summary feedback per balance exercise. A 5–10-min warm-up and a 5-min cool-down marked the start and end of each session. In between, 5–7 balance exercises (3 sets of 30–40 s per exercise) addressing static (i.e., standing exercises), dynamic (i.e., walking exercises), proactive (i.e., weight shifting while standing), and reactive (i.e., perturbed standing) balance were performed. In accordance to Granacher et al. [7], progression during BT was achieved by altering the visual input (e.g., from eyes opened to closed), by using unstable devices (e.g., soft mat, ankle disk, balance board, air cushion), and by reducing the base of support (i.e., from two-legged over step and tandem to one-legged stance).

#### Statistical analysis

Descriptive statistics were presented as group means ± standard deviations. Normal distribution was examined using the Shapiro–Wilk test (*p* > 0.05) and homogeneity of variances using the Levene test (*p* > 0.05). A 2 (group: BT-4wk, BT-6wk) × 2 (test: pre, post) analysis of variance (ANOVA) with repeated measures on test was used to detect differences during pre- and post-training testing. Post-hoc tests with the Bonferroni-adjusted *α* were conducted to identify comparisons that were statistically significant (i.e., *p* < 0.05). Further, the partial eta-squared (*η*_p_^2^) was calculated and classified as small (0.02 ≤ *η*_p_^2^ ≤ 0.12), medium (0.13 ≤ *η*_p_^2^ ≤ 0.25), and large (*η*_p_^2^ ≥ 0.26) [8]. All analyses were performed using the SPSS version 27.0.

### Results

#### Static balance performance

The ANOVA showed a significant main effect of test while standing on foam (*F*_(1, 27)_ = 4.605, *p* = 0.041, *η*_p_^2^ = 0.15) but not on firm ground (Table 2). The main effect of group and the group × test interaction did not reach the level of significance.

**Table 2:** Effects of balance training duration and volume on measures of static and dynamic balance performance in healthy children (*N* = 29)

Outcome	BT-4wk (*n* = 15)		BT-6wk (*n* = 14)		*p*-value (*η*_p_^2^)		
	Pretest	Posttest	Pretest	Posttest	Test	Test × Group	Group
*Static balance*							
OLS floor contacts, FI-EO [n]	0.47 ± 1.36	0.33 ± 0.72	0.21 ± 0.58	0.07 ± 0.27	.335 (.04)	.973 (.01)	.366 (.03)
OLS floor contacts, FO-EO [n]	1.47 ± 1.90	0.80 ± 1.32	1.50 ± 2.90	0.43 ± 0.76	.041 (.15)*	.621 (.01)	.779 (.01)
*Dynamic balance*							
AT reach distance [% LL]	78.2 ± 5.8	81.1 ± 5.7	76.8 ± 11.9	82.0 ± 17.6	.038 (.15)*	.527 (.02)	.945 (.01)
PM reach distance [% LL]	113.4 ± 9.0	115.2 ± 9.0	112.0 ± 14.4	117.7 ± 18.6	.088 (.10)	.368 (.03)	.905 (.01)
PL reach distance [% LL]	112.3 ± 10.8	111.5 ± 11.6	112.8 ± 10.7	113.5 ± 12.8	.996 (.01)	.529 (.02)	.768 (.01)
CS [% LL]	101.3 ± 7.1	102.6 ± 7.3	100.5 ± 11.4	103.5 ± 11.8	.085 (.11)	.384 (.03)	.895 (.01)

Data are presented as mean value ± standard deviation. Figures in brackets are effect sizes. * indicates post-hoc significance

*AT* anterior, *BT* balance training, *CS* composite score, *EO* eyes open, *FI* firm ground, *FO* foam ground (i.e., balance pad), *LL* leg length, *n* number of floor contacts, *OLS* one-legged stance, *PL* posterolateral, *PM* posteromedial

#### Dynamic balance performance

The ANOVA yielded a significant main effect of test for the AT direction (*F*_(1, 27)_ = 4.765, *p* = 0.038, *η*_p_^2^ = 0.15) only (Table 2). The main effect of group and the group × test interaction again did not reach the level of significance.

### Discussion

In accordance with our first hypothesis stating that both BT programs will result in enhanced postural control, the two groups showed partially significant improvements (medium effect sizes) of their static (reduced number of ground contacts during OLS on foam ground) and dynamic (increased YBT-LQ anterior reach distance) balance performance. This finding corresponds with those from previous original studies examining the effect of BT in youth [2–4]. Thus, the results of the present study together with those from the current literature (for a review see Gebel et al. [1]) indicate that BT is effective to enhance static and dynamic balance in healthy children.

In contrast to our second hypothesis assuming that a longer and more voluminous exposure to the balance demanding training stimuli would lead to greater improvements, no significant group by test interactions were detected. This indicates that four (i.e., 240 min of training) compared to six (i.e., 360 min of training) weeks of BT did not result in group-specific improvements. Our finding is in line with the results of the systematic review with meta-analysis conducted by Gebel et al. [1]. The authors calculated medium- and small-sized improvements in balance following four and six weeks of BT, respectively but did not find significant differences between the two training durations/volumes. What are likely reasons that the effectiveness of BT in children did not depend on training duration and volume? First, participants of the BT-4wk already achieved average (50–60th percentile) or above-average (70–80th percentile) YBT-LQ scores [9] at the posttest. Therefore, despite training progression, a ceiling effect could have already occurred after four weeks of BT and two further weeks of training could have had no additional effect. Second, the resulting difference between the 4-week (240 min in total) and the 6-week (360 min in total) exposure to the training stimuli of 120 min might be too little to elicit significantly greater adaptations. Therefore, future studies should examine whether the assumed added value of a longer and more voluminous training exposure becomes apparent after, for example, eight or twelve instead of four weeks of BT. Third, there is evidence that in children the postural control systems (e.g., vestibular, somatosensory, and visual system) are not yet fully matured [10, 11]. However, the full maturation of these systems seems to be a prerequisite for both, responding effectively to training stimuli and being sensitive to their application for different periods of BT. Consequently, it should be investigated whether the expected additional value of prolonged training exposure applies to individuals with fully mature postural control systems (e.g., adolescents).

### Conclusion

The present study investigated the effects of BT conducted for four compared to six weeks on static and dynamic balance performance in healthy youth. Partially, significant improvements in static (i.e., reduced number of ground contacts during OLS on foam ground) and dynamic (i.e., increased YBT-LQ anterior reach distance) balance were detected. However, the performance enhancements did not differ significantly with respect to the applied BT duration and volume. These findings indicate that BT is an effective training regimen in healthy children but a longer, more voluminous (360 min during 6 weeks) versus a shorter, less voluminous (240 min during 4 weeks) BT does not seem to give any additional value.

## Limitations

- Both the duration and the volume of BT differed between the two groups. Therefore, the observed effects cannot be attributed to either BT modality.
- Children who do not have a fully mature postural control system have been studied.

## Data Availability

The data generated and analysed during the present study are available from the corresponding author upon reasonable request.

## Abbreviations

ANOVA: Analysis of variance
AT: Anterior
BT: Balance training
CS: Composite score
EO: Eyes open
FI: Firm ground
FO: Foam ground (i.e., balance pad)
LL: Leg length
OLS: One-legged stance
PL: Posterolateral
PM: Posteromedial
YBT-LQ: Lower Quarter Y-Balance Test
